# Upregulated integrin α11 in the stroma of cutaneous squamous cell carcinoma promotes skin carcinogenesis

**DOI:** 10.3389/fonc.2022.981009

**Published:** 2022-08-08

**Authors:** Guillermo A. Martínez-Nieto, Hanna-Riikka Teppo, Noora Petrelius, Valerio Izzi, Raman Devarajan, Tiina Petäistö, Hengshuo Liu, Kris S. Kim, Sanna-Maria Karppinen, Heli Ruotsalainen, Jarkko Koivunen, Joni M. Mäki, Gilbert C. Walker, Taina Pihlajaniemi, Donald Gullberg, Ritva Heljasvaara

**Affiliations:** ^1^ ECM-Hypoxia Research Unit, Faculty of Biochemistry and Molecular Medicine, University of Oulu, Oulu, Finland; ^2^ Cancer Research and Translational Medicine Research Unit, University of Oulu, Oulu, Finland; ^3^ Medical Research Center, Oulu University Hospital and University of Oulu, Oulu, Finland; ^4^ Department of Pathology, Oulu University Hospital, Oulu, Finland; ^5^ Research Unit of Biomedicine, University of Oulu, Oulu, Finland; ^6^ Finnish Cancer Institute, Helsinki, Finland; ^7^ Matrix Biology Group, Department of Biomedicine, Centre for Cancer Biomarkers, University of Bergen, Bergen, Norway; ^8^ Department of Chemistry, University of Toronto, Toronto, ON, Canada

**Keywords:** cancer-associated fibroblast, extracellular matrix, collagen, lysyl oxidase, myofibroblast, DMBA/TPA, tumor microenvironment

## Abstract

Integrin α11β1 is a collagen-binding integrin that is needed to induce and maintain the myofibroblast phenotype in fibrotic tissues and during wound healing. The expression of the α11 is upregulated in cancer-associated fibroblasts (CAFs) in various human neoplasms. We investigated α11 expression in human cutaneous squamous cell carcinoma (cSCC) and in benign and premalignant human skin lesions and monitored its effects on cSCC development by subjecting α11-knockout (*Itga11^−/−^*) mice to the DMBA/TPA skin carcinogenesis protocol. α11-deficient mice showed significantly decreased tumor cell proliferation, leading to delayed tumor development and reduced tumor burden. Integrin α11 expression was significantly upregulated in the desmoplastic tumor stroma of human and mouse cSCCs, and the highest α11 expression was detected in high-grade tumors. Our results point to a reduced ability of α11-deficient stromal cells to differentiate into matrix-producing and tumor-promoting CAFs and suggest that this is one causative mechanism underlying the observed decreased tumor growth. An unexpected finding in our study was that, despite reduced CAF activation, the α11-deficient skin tumors were characterized by the presence of thick and regularly aligned collagen bundles. This finding was attributed to a higher expression of TGFβ1 and collagen crosslinking lysyl oxidases in the *Itga11^-/-^* tumor stroma. In summary, our data suggest that α11β1 operates in a complex interactive tumor environment to regulate ECM synthesis and collagen organization and thus foster cSCC growth. Further studies with advanced experimental models are still needed to define the exact roles and molecular mechanisms of stromal α11β1 in skin tumorigenesis.

## Introduction

The dynamic interactions of tumor cells with the surrounding tumor microenvironment (TME), consisting of various stromal cells, soluble factors, and the extracellular matrix (ECM), critically regulate all steps of tumorigenesis (1, 2). The non-vascular, non-inflammatory stromal cells within the TME are designated cancer-associated fibroblasts (CAFs) (3). Recent single-cell RNA sequencing (scRNA-seq), proteomics and flow cytometry approaches have revealed extraordinary heterogeneity and plasticity among CAFs subpopulations (2–4). The three major subtypes of CAFs, originally identified in pancreatic adenocarcinoma (PDAC), are myofibroblastic CAFs (myCAFs), inflammatory CAFs (iCAFs), and antigen-presenting CAFs (apCAFs) (5, 6). The biomarkers expressed in the different subsets of CAFs include alpha-smooth muscle actin (αSMA), fibroblast-specific protein 1 (FSP-1), platelet-derived growth factor receptors α and β (PDGFRα/β), and fibroblast-activating protein (FAP). CAFs are often characterized by the synthesis of ECM proteins, including fibrillar collagens, proteoglycans, matricellular proteins, and ECM-modifying enzymes, contributing to tumor fibrosis (2, 7). A current challenge in TME research involves identifying, characterizing, and targeting tumor-promoting CAF subtypes (2–4).

Cell-ECM interactions and signalling through αβ heterodimeric integrin receptors regulate the properties and functions of tumor cells, as well as the various cell types found in the TME. Tumor stromal integrins regulate tumor stiffness, matrix reorganization, tumor angiogenesis, CAF activation, and metastasis (8–10). The vertebrate integrin family is composed of 18 α subunits and 8 β subunits which can form 24 different αβ heterodimers. Integrin α11β1, together with α1β1, α2β1, and α10β1, constitute a subgroup that can bind collagens and associate exclusively with the β1 subunit (11). The first report demonstrating the importance of fibroblast-derived integrin α11 in cancer focused on non-small-cell lung cancer (NSCLC) and showed that α11β1 is highly expressed in the tumor stroma and promotes tumorigenesis through the induction of insulin growth factor 2 expression in CAFs (12). Later studies on NSCLC demonstrated that α11β1 signaling leads to the upregulation of lysyl oxidase like-1 (LOXL1) and, subsequently, increased collagen crosslinking, stromal stiffness, and tumor growth and progression (13, 14). High α11 expression in CAFs has been reported in many other solid tumors, such as head and neck squamous cell carcinoma (HNSCC) and breast cancer and PDAC, in which it is associated with a poor prognosis (15–17). Mechanistically, α11β1 has been shown to regulate the PDGFRβ/JNK signaling axis in breast cancer CAFs, leading to ECM remodeling and CAF-induced tumor cell invasion (16). In PDAC *in vitro* systems, the knockdown of α11 in pancreatic stellate cells inhibited cell contractility, migration, and differentiation and reduced the expression of several ECM components (17).

Cutaneous squamous cell carcinoma (cSCC) is the second most common human cancer. It is principally caused by ultraviolet light, increasing the incidence of this cancer type in sun-exposed areas of the body (18, 19). Although early detection and surgery can prevent complications, primary cSCC can frequently recur and metastasize, with an 5% average rate of metastasis. The accumulation of genetic mutations in epithelial cells fosters the progression of the disease from precancerous actinic keratosis lesions to cSCC *in situ*, to the invasive form of cSCC and, finally, to metastatic cSCC. The stromal compartment plays an important role in cSCC progression, and changes in ECM composition and properties, as well as inflammatory and immune cells, contribute to a milieu that favors tumorigenesis (20, 21).

The expression of β1-integrins and their ligands is known to correlate with tumor progression in human cSCC, and the roles of major epidermal β1 integrins (α2β1, α3β1) are widely studied (19, 22). In contrast, the data regarding the functions and significance of β1-integrins in CAFs are still limited (23). To advance our understanding of integrin α11β1 in cSCC, we analyzed the expression of the α11 subunit in human cSCC samples and in benign and premalignant human skin lesions *via* immunohistochemistry (IHC) and subjected mice deficient in the α11 subunit to the broadly used murine model of skin cancer, the multistep chemical skin carcinogenesis protocol involving 7,12-dimethylbenz[α]anthracene (DMBA) and 12-*O*-tetradecanoylphorbol-13-acetate (TPA) treatments (24). Our studies demonstrate, for the first time, the upregulation of integrin α11 in CAFs in human and mouse cSCC, as well as its tumor-promoting role in an experimental mouse model of skin carcinogenesis. We also describe interesting alterations in the skin tumor stroma on account of α11 ablation in mice.

## Materials and methods

### Human samples and immunohistochemistry

Formalin-fixed, paraffin-embedded (FFPE) human skin tissue samples were collected from the archives of the Department of Pathology, Oulu University Hospital, Finland, and consisted of 19 seborrheic keratosis samples, 14 actinic keratosis samples, five squamous carcinomas *in situ* (also known as Bowen’s disease), 15 keratoacanthoma samples, and 29 cSCC samples (total n = 82). Diagnoses were made according to the current WHO classification (25). The use of skin specimens was approved by the Finnish National Supervisory Authority for Welfare and Health (V/12456/2019) and the Ethical Committee of the Northern Ostrobothnia Hospital District (Dnr. 100/2018). Studies were carried out in accordance with the provisions of the Helsinki Declaration (1983).

Skin tissues were stained with a previously validated monoclonal anti-human integrin α11 antibody (clone 210F4B6A4) (26) using an Envision FLEX+ kit (Dako, K800221-2). 3.5 µm-thick FFPE sections were dried at 55°C for 48h, deparaffinized in xylene (3 min, 3 times), and rehydrated through graded ethanol solutions. Antigen retrieval was performed using Tris-EDTA pH 9 (EnVision FLEX Target Retrieval Solution, high pH; Dako, K801021-2) by boiling with microwaves at 850W for 2 min and 150W for 15 min. After boiling, the sections were allowed to cool at room temperature (RT) and washed using distilled water and EnVision FLEX Wash Buffer for 10 min. The sections were incubated in the peroxidase blocking solution of the kit for 8 min and washed with EnVision FLEX Wash Buffer for 10 min. After washing, the sections were incubated with the primary α11 antibody for 1 h at RT at a 1:1000 dilution following overnight incubation at +4°C, incubated again at RT for 60 min, and then washed with EnVision FLEX Wash Buffer for 10 min. Then, the samples were incubated with secondary antibody (EnVision FLEX+ mouse linker) and visualized using EnVision FLEX/HRP and DAB according to the manufacturer’s instructions. After being rinsed with distilled water, sections were counterstained with hematoxylin, rinsed, dehydrated, cleared, and mounted. The samples were imaged using a Hamamatsu NanoZoomer S60 whole-slide digital scanner in the 20× mode. In dermal spindle-shaped cells, the staining intensity was evaluated as one of the following expressions: absent (0), weak (1), moderate (2), or strong (3). The quantity of each intensity level was recorded:<10% (1), 10–50% (2), or >50% (3). Subsequently a staining index (SI) score (0–9) was obtained by multiplying the score for intensity by the quantity percentage group. The unambiguous α11 signal in myoepithelial cells around the sweat glands served as a positive control. Hematoxylin-eosin (H&E)-stained samples were used for cSCC grading.

### Mice

Integrin-α11-knockout mice (*Itga11^tm1Dgul^*, MGI:3714472) (27) were backcrossed to the FVB/N (Harlan, The Netherlands) strain for at least five generations. The mice were maintained in a pathogen-free facility, group-housed with corncob bedding and enrichments at 21°C with a 12:12-hour light:dark cycle and given *ad libitum* water and standard rodent chow. Mice were maintained and animal experiments were conducted in the Laboratory Animal Centre of the University of Oulu (OULAC), following the the regulations for the protection of vertebrate animals used for experimental and other scientific purposes (European Convention Treaty ETS No. 23, European Community Council Directive 2010/63/EU, and Finnish Government Decree 564/2013).

### Chemical skin carcinogenesis and hyperplasia models

Skin carcinogenesis experiments were approved by the National Animal Experiment Board of Finland (license ESAVI/1188/04.10.07/2016). Skin tumors were induced in seven-week-old *Itga11^+/+^* (n = 25) and *Itga11^-/-^* (n = 21) male mice with topical DMBA (Sigma-Aldrich) and TPA (Sigma-Aldrich) treatments (24). The dorsal skin of the mice was shaved and treated with a single dose of DMBA (100 μg in 100 μl acetone), followed by weekly TPA treatments (5 μg in 100 μl acetone), which continued until the mice were removed from the experiment at predetermined time points (15, 20, 25, and 30 weeks) or on the basis of humane endpoints (engraved or ulcerative cSCCs, tumors with a diameter over 10 mm, or excessive tumor load per mouse). During the treatments and tumor monitoring, the mice were housed in single cages to avoid fights and skin wounding, which can have an impact on tumorigenesis. Tumor growth was monitored once per week, and tumor number, size and macroscopic appearance were recorded. Tumor incidence (percentage of mice with a tumor) and cumulative tumor multiplicity (number of papillomas divided by the total number of mice alive at the time when the first mouse was removed from the experiment because of humane endpoints) were recorded and tumors were dissected for further analyses. H&E-stained carcinoma samples were analysed and graded by a pathologist in a blinded manner. To label the actively proliferating tumor cells, 100 µg/kg of 5-bromo-2-deoxyuridine (BrdU, Abcam) was injected intraperitoneally into the mice two hours before sacrifice.

To induce epidermal hyperplasia, the shaved dorsal skin of seven-week-old male mice was treated six times, at two-day intervals, with 5 µg of TPA in acetone, and skin samples were collected for analyses. Mouse skin treated with acetone was used as a control.

Mice were euthanized with CO_2_ inhalation and cervical dislocation. Tissues were dissected and fixed in fresh phosphate-buffered 4% paraformaldehyde (PFA) for 24 hours at +4°C, washed for one hour under running tap water, dehydrated in ethanol of increasing concentration, cleared with xylene, and embedded in paraffin. Alternatively, the dissected tissues were embedded in OCT compound (Tissue-Tek, Sakura Finetechnical) and frozen for cryo-sectioning.

### Immunofluorescence staining

Immunofluorescence was used to visualize integrin α11-, Ki67-, cytokeratin 5- (CK5), NG2-, PDGFRβ-, LOX-, and αSMA-positive cells in 5 µm-thick cryosections (**Supplementary Table S1**). Staining with rabbit polyclonal anti-integrin α11 antibody (1:200 dilution) (13, 28) was performed overnight at +4°C, after a 10 min methanol-acetone fixation at −20°C and blocking with 10% goat serum in PBS for one hour at RT. For monoclonal anti-Ki67 antibody ethanol-fixed (10 min at −20°C) cryo-sections were used, and antigen retrieval with 1% Triton-X and blocking with goat 10% serum for one hour at RT preceded the overnight incubation with the primary antibody +4°C. For the CK5, NG2, PDGFRβ, LOX, and αSMA antibodies, the blocking solution contained 1% BSA and 22.5 mg/ml glycin in PBS/0.1% Tween. Appropriate goat anti-rabbit Alexa Fluor 488 (1:300, Invitrogen) and goat anti-rat or donkey anti-mouse Cy3 (1:200, Jackson ImmunoResearch) secondary antibodies were used for the detection. To visualize αSMA in PFA-fixed tissues by immunofluorescence, antigen retrieval was accomplished by treating the 5 µm-thick sections in Tris/EDTA in boiling water for 10 min, followed by blocking in 10% goat serum for one hour at RT and overnight incubation with Cy3-conjugated anti-αSMA antibody. To detect BrdU, sections were blocked with 5% donkey serum, treated with 2M HCl (30 min) and 0.1M sodium borate (10 min), and then stained with anti-5-Bromo-2-Deoxyuridine antibody, followed by the application of donkey anti-mouse Cy3 (1:200, Jackson ImmunoResearch). Cell nuclei were labelled with DAPI (1:300, Sigma-Aldrich). A confocal microscope Zeiss LSM 700 or 780 was used for imaging. The intensities of the fluorescent PDGFRβ, αSMA, and LOX signals in the tumors were quantified from 20× confocal microscopy images in a blind manner using Fiji ImageJ analysis software.

### Analysis of collagen content

To analyze the content of fibrillar collagen in skin tumors, Masson trichrome and picrosirius red stainings were performed as described previously (29). PFA-fixed tumor sections were dewaxed in xylene; rehydrated with decreasing ethanol series; and treated with a nuclear stain (Celestine blue and Harris’ hematoxylin), acid fuchsin, phosphomolybdic acid, and methyl blue stain (all reagents from Sigma–Aldrich). For picrosirius red staining, PFA-fixed tumor sections were treated with 0.2% phosphomolybdic acid for 5 min, followed by staining with 0.1% Direct Red 80/Sirius Red F3B (Sigma–Aldrich) in saturated picric acid for 1 h at RT. Both protocols were finished *via* dehydration with an ethanol series, clearing with xylene, and mounting with Pertex (Sigma–Aldrich). The Masson’s trichrome-stained sections were imaged with a Leica DM LB2 microscope, and picrosirius red-stained sections were imaged under bright and polorized light with an Olympus BX51 microscope (Olympys, Tokyo, Japan). Fiji ImageJ software was used for birefringence signal quantification, and the ratio of thick versus thin collagen fibers was calculated as described previously (30).

### Transmission electron microscopy

Papillomas were dissected from mice; cut into 1 mm^3^ cubes; fixed with 1% glutaraldehyde, 4% formaldehyde, and 0.1 M phosphate buffer (pH 7.4) for 12 hours at RT; post-fixed in 1% osmium tetroxide for 15 min; dehydrated in acetone; and embedded in Epon LX 112 (Ladd Research Industries). 80 nm sections were examined using a Tecnai G2 Spirit transmission electron microscope, and images were captured using Veleta or Quemesa CCD cameras (Olympus Soft Imaging Solutions). A total of six papillomas from different individuals, three per genotype, were analyzed using TEM.

### Fluorescence-activated cell sorting

Mouse skin pieces were dissected and digested in 0.25% collagenase I (w/v) in DMEM for 2 hours at +37°C, disaggregated with vigorous pipetting, and passed through a 40 μM cell strainer. The resulting single-cell suspension was analyzed for different cell populations using FACSCalibur running the CellQuest Pro software (BD Biosciences). Fibroblast and immune cell populations were analysed with antibodies listed in **Supplementary Table S2**. Data were examined using FlowJo software (FlowJo LLC).

### *In vitro* adipogenesis assay

Mesenchymal (MSC) progenitors were isolated from the stromal vascular fraction (SVF) of the mouse inguinal fat pads and differentiated into mature adipocytes using a standard protocol (31, 32), with minor modifications. Six-week-old mice were euthanized, sprayed with ethanol, and opened in a laminar hood. Inguinal fat pads were collected from four to five mice per genotype and pooled in cold phosphate buffered saline (PBS). Fat tissues were minced and digested with 2.5 mg/ml of collagenase D (Roche) and 3.1 U/ml of Dispase II (Sigma-Aldrich) in 10 mM CaCl_2_/PBS for 45 min at 37°C with regular shaking. Digestion was terminated by adding complete preadipocyte medium consisting of DMEM/F12 supplemented with 10% FBS, 1% penicillin/streptomycin and 0.1mg/ml primocin (*In vivo*Gen). The cellular suspension was filtered through 100 μm cell strainer to remove undigested tissue debris and centrifuged at 600 x g for 5 min at +4°C. SVF cells were suspended in preadipocyte medium, the suspension was filtered through a 40 μm cell strainer, and cells were seeded on a 10 cm culture plate in the complete preadipocyte medium. On the next day, the cells were washed four times with PBS and then cultured in complete preadipocyte medium. The culture medium was changed every day until the cells reached 90% confluence. Cells were divided and allowed to grow to confluence, and two days post-confluence, adipocyte differentiation was induced by adding preadipocyte medium supplemented with 5µg/ml insulin (Sigma-Aldrich), 1µM rosiglitazone (Cayman), 0.5mM isobutylmethylxanthine (Sigma-Aldrich), and 1µM dexamethasone (Sigma-Aldrich). After 48 h of induction, the cells were maintained in medium containing 1µM rosiglitazone and 5µg/ml insulin, and cell differentiation was monitored for 6 days, changing the maintenance medium daily. Cell samples were collected for RNA analyses every two days, and Oil Red-O staining was performed at the end of the experiment to measure to lipid accumulation. The medium was collected on Day 6 to measure the glycerol concentration. Cells were imaged using the Olympus CellSens imaging system with a 10**×** objective and supplemented with the Olympus XM10 CCD camera (Tokyo, Japan). The adipogenesis assay was repeated with at least three primary cell preparations per genotype.

### Quantitative real-time polymerase chain reaction

Total RNA was extracted from snap-frozen mouse tumors and hyperplastic skin with TriPure reagent (Roche), followed by the RNeasy mini kit (Qiagen). A total of 0.5 μg RNA, pooled from at least five papillomas collected from five individuals of each genotype at a selected time point, was used to synthesize cDNA by using the iScript cDNA synthesis kit (Bio-Rad). Samples were analysed for the mRNA expression of α1 chains of collagen I (*Col1a1*) and III (*Col3a1*), prolyl 4-hydoxylases-1 (*Pdha1*) and -2 (*Pdha2*), αSMA (*Acta2*), PDGFRα (*Pdgfra*), PDGFRβ (*Pdgfrb*), and tenascin-C (*Tnc*). The PCR primers are listed in **Supplementary Table 3**. The qPCR was performed with iTaq SYBR Green Supermix with ROX reagents (BioRad), and each sample was run in duplicate by using a Mx3005P qPCR device (Stratagene) and repeated at least three times. Relative mRNA levels were calculated with the 2^ΔΔCt^ method (33). Values were normalized against *Gapdh*, and the control values were expressed as 1 to indicate the fold change in mRNA expression.

### Atomic force microscopy

Force-distance curve measurements were collected using an MFP-3D Molecular Force Probe AFM (Asylum Research, Santa Barbara) and a borosilicate glass particle (5 μm in diameter) on silicon nitride cantilevers (Novascan Tech Inc., Ames). The cantilevers had a nominal spring constant of 0.02 N/m and were calibrated before each experiment with the thermal noise method (34). All measurements were collected in 18.2 MΩ cm Milli-Q water. Each AFM probe was used to measure one control and one knockout sample, and the order of sample measurement was randomized for each probe. The samples were indented at a loading rate of 500 nm/s with a maximum force of 1 nN. 300 force extension curves were collected over three randomly chosen 20 μm × 20 μm areas of each sample. The results show the contributions of four *Itga11^+/+^* and three *Itga11^-/-^* samples. The determination of the Young’s elastic modulus of the samples from force-distance curves was performed using the Hertz model (35, 36).


$$F=\frac{4\sqrt{R}}{3\left(1-\nu^{2}\right)}E\delta^{3/2}$$

where *F* is the loading force (N), E is the Young’s modulus (Pa), *v* is the Poisson ratio, *R* is the radius of curvature of the tip (m), and δ is the indentation depth (m). Samples were assumed to be incompressible, and a Poisson’s ratio of 0.5 was used in the calculation of the Young’s elastic modulus.

### Statistical analysis

Statistical significance was computed with a two-tailed unpaired *t-*test or ANOVA using Prism software. The differences were considered significant at p< 0.05 and expressed in figures as *, p< 0.05; **, p< 0.01; *** and p< 0.001. Figure data are presented as means ±SEM or ±SD, as indicated in the respective figure legends. For human IHC, statistical analyses were performed by using IBM SPSS Statistics software, Version 28.0.0.0 (IBM Corporation, Armonk, NY, USA). The significance of associations was defined by using the Mann-Whitney *U* test and Kruskal-Wallis test.

## Results

### Integrin α11 is upregulated in the stroma of human and mouse cSCC

**Figure 1:**
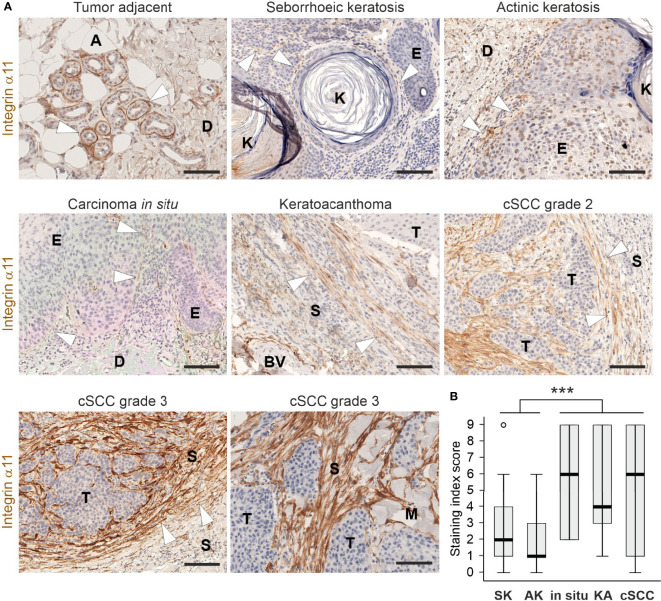
Expression and localization of integrin α11 in human cutaneous lesions. **(A)** Representative images of integrin α11 expression and localization in human skin lesions, stained with a monoclonal anti-human integrin α11 antibody (clone 210F4B6A4) (26). Integrin α11 showed strong expression around sweat glands in the tumor-adjacent normal skin, likely in the myoepithelial cells of the acini (arrowheads); scant positive signals in spindle-shaped cells at the dermal-epidermal junction (arrowheads) in benign seborrheic keratosis, premalignant actinic keratosis, and in squamous carcinoma *in situ*; generally moderate or strong signals in spindle-shaped cells distributed within the fibrillar stroma; and present in a tangle-like pattern at the tumor−stroma interphase (arrowheads) in malignant keratoacanthoma and cutaneous squamous cells carcinomas (cSCC). Stromal α11 staining is strong in grade 3 cSCCs. **(B)** A boxplot diagram representing the staining index score in seborrhoeic keratosis (SK), actinic keratosis (AK), *in situ* carcinomas (in situ), keratoacanthomas (KA), and cSCCs. Arrowheads, α11 signals. A, adipocyte, BV, blood vessel; D, dermis; E, epidermis; K, keratin; M, muscle; S, stroma; T, tumor. Scale bars, 100 μm. ***, p<0.001.

Integrin α11 expression and localization were examined in a collection of pre-malignant and malignant human skin lesions using IHC and a previously validated monoclonal anti-human integrin α11 antibody (26). In the benign seborrheic keratosis and premalignant actinic keratosis specimens, as well as squamous carcinoma *in situ*, weak or moderate membranous α11 signals were regularly detected in spindle-shaped cells at the dermal-epidermal junction (**Figure 1A**). In the malignant keratoacanthoma and cSCCs, α11 expression showed upregulation but also considerable variation. Often, the α11-positive cells were located diffusely throughout the tumor stroma, the intensity of staining ranging from weak to strong, as well as being absent in some cases. Intense α11 staining was frequently seen as a fibrillar, tangle-like pattern at the tumor-stroma interphase. The number of α11-positive cells varied substantially, even within a given tumor sample (**Figure 1A**; **Supplementary Figure 1**). The samples with the strongest staining were classified as grade-3 cSCCs (**Figure 1A**). The quantification of α11 expression in skin lesions using an SI score (*i.e.*, the number of α11-positive spindle-like cells and staining intensity) showed high variability within the diagnostic groups of malignant cases (**Figure 1B**). Nevertheless, SI scores differed significantly between the groups of benign to premalignant keratoses and the groups of malignant carcinomas (p<0.001), demonstrating that the stromal upregulation of α11 integrin is associated with malignancy in human cSCC. No membranous α11 staining was observed in tumor cells, but the nuclear positivity of keratinocytes was frequently observed. Because we have not observed α11 RNA in keratinocytes (37), this signal may be an artefact related to heat-induced antigen retrieval at a high pH (38). In tumor-adjacent tissues, prominent α11 signals were also localized to the myoepithelial cell layer of the sweat glands and around the hair follicles (**Figure 1A**; **Supplementary Figure 1**).

**Figure 2:**
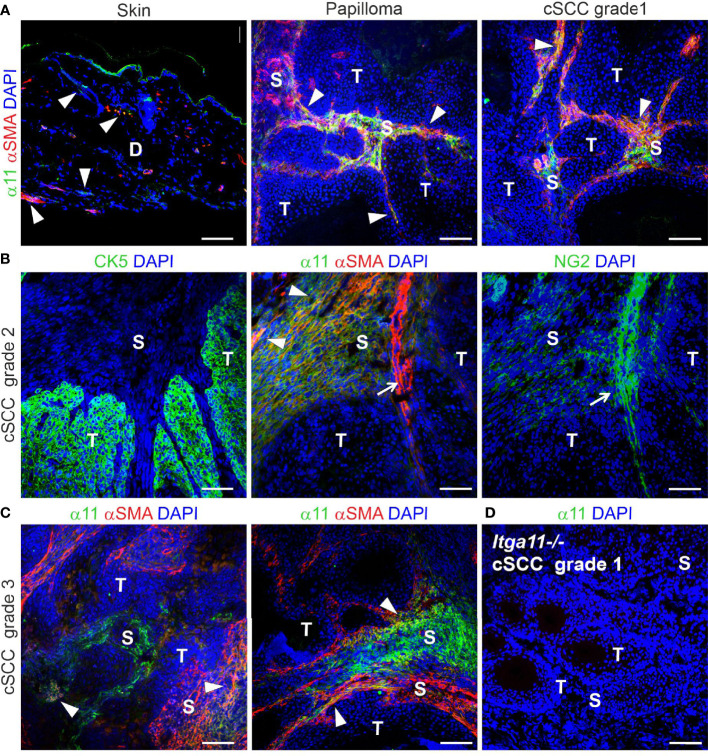
Expression and localization of integrin α11 in mouse skin and chemically induced skin tumors. Representative images of α11 immunofluorescence in normal mouse skin and skin tumors, stained with a polyclonal anti-mouse α11 antibody (28). **(A)** α11 expression is negligible in normal mouse skin; scant positive signals are found around hair follicles and in isolated dermal cells (arrowheads), sometimes overlapping with smooth muscle actin (αSMA) signals. The positive signal in the cornified epithelium represents non-specific tissue autofluorescence due to Alexa Fluor 488-conjugated secondary antibody. **(A-C)** α11 is significantly upregulated in the stroma of premalignant papillomas and malignant cSCCs of different grades and partially co-localizes with αSMA-positive cells (arrowheads). **(B)** Sequential sections of a moderately differentiated (grade 2) cSCC. Integrin α11 and αSMA are localized exclusively in the tumor stroma and show partial overlapping (arrowheads). αSMA is also detected in smooth muscle cells around some blood vessels (arrow) and is closely associated with a pericyte marker, NG2. Cytokeratin 5 (CK5) is a marker of carcinoma cells and does not co-localize with α11. **(C)** Examples of integrin α11 and αSMA staining in grade 3 cSCC; immunofluorescent signals in the stroma occasionally overlap with αSMA (arrowheads). **(D)** A well differentiated (grade 1) cSCC from a *Itga11^-/-^* mouse was used as a staining control for the α11 antibody. Scale bars: A, 100 μm, B-D, 50 μm. Markings in images: D, dermis; S, stroma; T, tumor.

In addition to human tissue material, we studied α11 expression and localization in healthy murine skin and in DMBA/TPA-induced skin tumors obtained from wild type FVB/N mice (39). In normal mouse skin, α11 expression was barely detected in the dermis. A significant upregulation of α11 was observed in mouse skin tumor samples, both in papillomas and in cSCCs of different grades, in which α11 was abundantly expressed in the tumor stroma (**Figures 2A-C**). Often but not always, α11 signals overlapped with αSMA, a common marker of myofibroblasts and myCAFs. The chondroitin sulphate proteoglycan NG2, a marker of pericytes and subsets of CAFs, showed faint signals in α11-positive stromal areas and prominent signals in the tumor vasculature, where it was associated with αSMA (**Figure 2B**). α11 expression was not detected in tumor areas, which were visualized by cytokeratin (**Figure 2B**). As in human cSCC, variation in α11 signal intensity and localization was observed between tumor samples and even different stromal regions of the same tumor. Thus, our immunostainings show that α11 is highly upregulated in both human and mouse cSCC stroma and localized in spindle-shaped cells, which, in mouse tumors, were shown to represent specific CAF subtypes. The data suggest a role for α11 in cSCC tumorigenesis and prompted us to subject α11-knockout (*Itga11^−/−^*) mice to an experimental skin carcinogenesis model.

### Skin tumor growth is impaired in integrin α11-deficient mice

**Figure 3:**
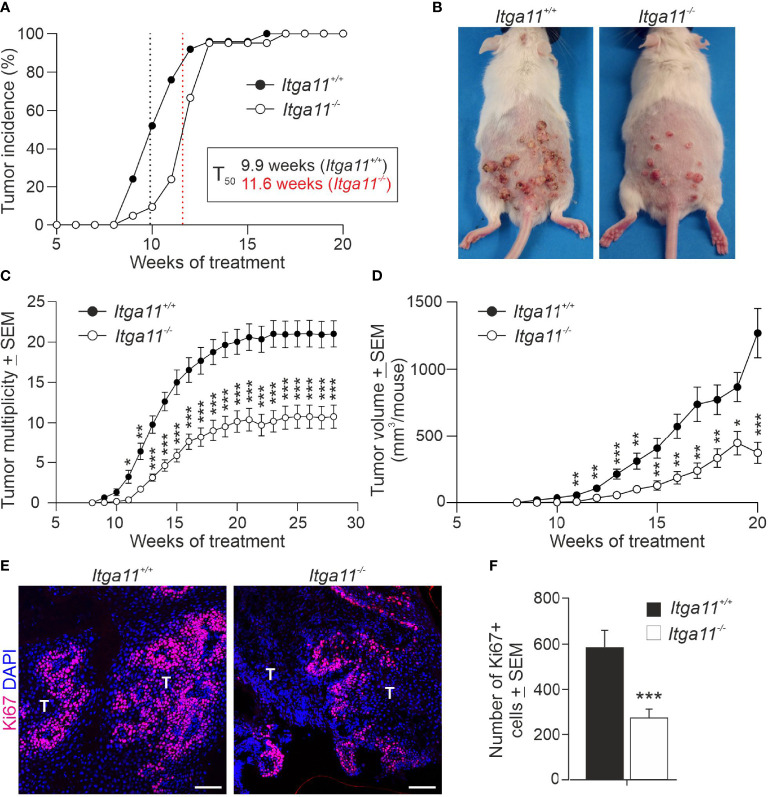
Skin tumor growth is impaired in integrin α11-deficient mice. Tumors were induced in the dorsal skin of the *Itga11^+/+^* (n = 25) and *Itga11^-/-^* (n = 21) male mice using the 7,12-dimethylbenz[a]anthracene (DMBA)/12-O-tetradecanoylphorbol-13-acetate (TPA) protocol, and tumor incidence and multiplicity were monitored for up to 28 weeks in some individuals. **(A)** Tumor incidence. At week 10, the tumor incidence in the *Itga11^-/-^* mice was approximately 50% that in the *Itga11^+/+^* mice. There was a delay of two weeks in terms of tumor incidence in the *Itga11^-/-^* mice (T_50_); however, all mice developed skin tumors by week 13. **(B)** Representative photographs of the DMBA/TPA-treated *Itga11^+/+^* and *Itga11^-/-^* mice at week 20. **(C)** Cumulative tumor multiplicity. Compared to the *Itga11^+/+^* controls, the *Itga11^-/-^* mice developed roughly 50% fewer skin tumors upon DMBA and TPA treatments. **(D)** The total tumor burden per mouse was significantly smaller in the *Itga11^-/-^* mice from week 10 onwards. **(E, F)** Tumor cell proliferation. Representative images of Ki67 staining of the control and α11-deficient skin papillomas and quantification of proliferating Ki67-positive cells. Ten papillomas per genotype (from different mice) and four to five microscopic fields for each papilloma sample at a magnification of 200× were counted. In E, scale bars 100 μm; T, tumor. *, p<0.05, **, p<0.01, ***, p<0.001.

To evaluate the relevance of integrin α11β1 integrin signaling in skin carcinogenesis, we compared the development and progression of DMBA/TPA-induced skin tumors between α11-deficient (*Itga11^−/−^*) and control mice (*Itga11^+/+^*) mice. The absence of α11 expression in knockout tumors was confirmed by immunofluorescence staining (**Figure 2C**). We observed a significant impairment in tumor growth in the *Itga11^−/−^* mice as compared with the controls. First, a delay of approximately two weeks in terms of tumor incidence (*i.e.*, the proportion of mice bearing at least one tumor) was observed in the *Itga11^−/−^* mice, with the difference being most evident at week 11, when approximately 75% of the control mice but only 25% of the *Itga11^−/−^* mice had developed measurable papillomas (**Figure 3A**). Impaired tumorigenesis in the *Itga11^−/−^* mice was seen even more clearly when the tumor multiplicity between these and the *Itga11^+/+^* controls was compared. A marked reduction in the number of skin tumors was apparent already at week 10, and at weeks 20–28, the *Itga11^−/−^* mice had, on average, 50% fewer papillomas than the *Itga11^+/+^* mice (**Figures 3B, C**). In addition, a difference in tumor size was observed between the genotypes such that, in the *Itga11^−/−^* mice, the papillomas were generally smaller than those in the controls (**Supplementary Figure 2A**). In consequence, the total tumor burden per mouse was significantly smaller in the *Itga11^−/−^* mice from week 10 onwards (**Figure 3D**).

Given the marked difference in tumor growth between the *Itga11^+/+^* and *Itga11^−/−^* mice, we addressed the impact of α11 in tumor cell proliferation by determining the numbers of actively dividing cells in tumor tissue sections. Anti-Ki67 staining showed a significant, up to 50%, reduction in the number of dividing tumor cells in *Itga11^−/−^* tumors relative to *Itga11^+/+^* tumors at week 20 (**Figures 3E, F**). *In vivo 5*-bromo-2’-deoxyuridine (BrdU) labelling and subsequent anti-BrdU staining confirmed this finding, showing a roughly 30% reduction of BrdU-positive cells in *Itga11^−/−^* tumors at the same time point (**Supplementary Figures 2B, C**). As expected, the numbers of proliferating cells were trivial in the untreated skin in both genotypes and were not counted, underpinning our observation that the upregulation of α11 expression in CAFs boosts tumor cell proliferation.

Only a small portion of papillomas progressed to invasive cSCCs in our experimental setup, and these typically presented as doughnut-shaped tumors with erosion and/or ulceration. Recording the incidence of tumors with these features at weeks 15–25 showed that, compared the to the *Itga11^−/−^* mice, the *Itga11^+/+^* mice had almost double the number of tumors with a malignant appearance (**Supplementary Table 4**). However, because the overall tumor number was considerably lower in the knockout mice, the calculated conversion rates were equal between the genotypes (**Supplementary Figure 2D**). When the dissected cSCC suspects were histologically graded, 64% (n = 9/14) of those in the *Itga1^+/+^* mice represented cSCCs of grades 1–3, and 36% (n = 5/14) represented benign or dysplastic papillomas, whereas in the *Itga11^−/−^* mice, 29% (n = 2/7) of tumors were eventually scored as cSCCs, and 71% were scored as (n = 5/7) as papillomas (**Supplementary Table 4**).

### Alterations in the ECM and immune microenvironments in *Itga11^−/−^* skin tumors

**Figure 4:**
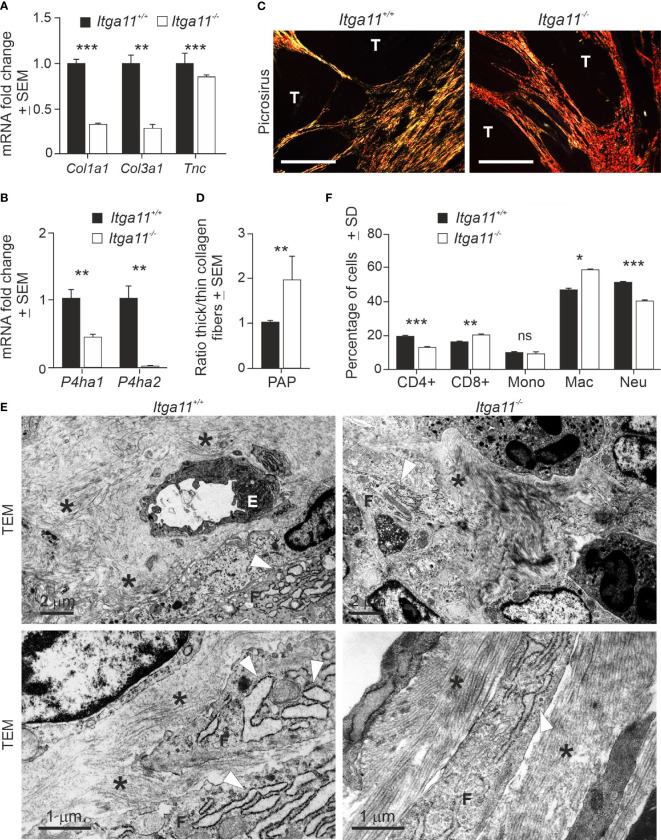
Characterization of α11-deficient of skin tumor stroma. **(A, B)** RT-qPCR analysis of collagen I (*Col1a1*) and III (*Col3a1*) α1 chain, tenascin (*Tnc*), and prolyl-4-hydoxylase 1 (*Pdha1*) and 2 (*Pdha2*) in *Itga11^+/+^* and *Itga11^-/-^* papillomas. RT-qPCR data are an average of ten samples (from different individuals) per genotype collected at weeks 19–20 and normalized with endogenous *Gapdh*. **(C)** Representative images of picrosirius red staining of *Itga11^+/+^* and *Itga11^-/-^* papillomas imaged by polarized light microscopy. Thin collagen fibers appear as yellow/green, and thick collagen bundles appear as red/orange tones. Scale bar, 200 μm. T, tumor. **(D)** The quantification of collagen birefringence shows that the ratio of thick/thin collagen fibers is significantly increased in the *Itga11^-/-^* tumors compared to the *Itga11^+/+^* tumors (n = 10 per genotype). **(E)** Transmission electron microscopy (TEM) of skin tumors (n = 3 per genotype). Representative images of two separate *Itga11^+/+^* and two *Itga11^-/-^* papillomas with different magnifications are shown. In the *Itga11^+/+^* papillomas, a prominent dilated rough endoplasmic reticulum (rER, arrowhead) of fibroblasts is evident, and collagen fibers (asterisk) are scattered in the stroma. In the *Itga11^-/-^* tumors, collagen fibers are organized parallelly in large bundles (asterisk), and the rER is less evident. E, endothelial cell; F, fibroblast; scale bars are marked in the pictures. **(F)** FACS analysis of immune cells in skin tumors. Ten papillomas per genotype, which were harvested from three individuals, were analyzed. In **(A, B, D, F)** *, p<0.05; **, p<0.01; ***, p<0.001. ns, not significant.

High integrin α11 expression has been associated with the matrix-producing and -remodeling CAF subpopulations, including the cells designated as mCAFs in breast cancer (40) and myCAFs in PDAC (41). Hence, we analyzed the expression of selected ECM components in skin tumors by qRT-PCR and found that the mRNA levels of α1 chains of collagen I (*Col1a1*) and III (*Col3a1*) and *Tnc* were significantly higher in *Itga11^+/+^* papillomas than in *Itga11^−/−^* papillomas (**Figure 4A**). The mRNA levels of prolyl 4-hydoxylases-1 (*Pdha1*) and -2 (*Pdha2*), the key enzymes in collagen biosynthesis, were also significantly downregulated in *Itga11^−/−^* papillomas (**Figure 4B**). Masson trichrome staining did not reveal obvious differences in the collagen content of tumors, although areas with hyalinized collagen, suggestive of thicker collagen fibers (42), were frequently observed in the *Itga11^−/−^* tumor stroma (**Supplementary Figure 3A**). Inspection of picrosirius red-stained tumor samples under polarized light showed that collagen fibers in *Itga11^−/−^* tumors often appeared orange or red, whereas they were yellow or green in *Itga11^+/+^* control tumors, indicating the presence of thicker collagen fibers in the knockout samples (**Figure 4C**). When the red and green signals were quantified, the ratio of thick/thin collagen fibers was approximately twofold higher in the *Itga11^−/−^* papillomas as compared with that in the *Itga11^+/+^* papillomas (**Figure 4D**).

The ultrastructural analysis of papillomas confirmed the differences in collagen fiber organization and also revealed potential differences in the activity of fibroblasts. In the α11-deficient tumors, the collagen fibers were organized in thick, regularly aligned bundles amongst the fibroblasts, whereas in the control tumors, the fibers were dispersed into scattered scaffolds (**Figure 4E**). The rough endoplasmic reticulum (rER) was prominent and highly dilated in the *Itga11^+/+^* tumor fibroblasts, whereas it was clearly less dilated in the *Itga11^−/−^* CAFs. An obvious, dilated rER is associated with myofibroblasts or CAFs in pathological conditions but not with resident (or less active) fibroblasts (43). Our data suggesting lower fibrillar collagen biosynthesis on the part of α11-deficient CAFs are congruent with the less active rER in these cells but stand in contrast with the observed well-organized collagen bundles in the *Itga11^−/−^* tumor stroma (**Figure 4E**).

We addressed the immune cell profiles in the TMEs of mouse skin tumors in order to determine whether α11-positive CAFs exerts paracrine effects on the immune environment, as well as whether these potential alterations could contribute to the observed impaired skin tumor growth in *Itga11* knockout mice. The FACS analysis showed that the *Itga11^−/−^* papillomas harbored significantly more macrophages and CD8^+^ T-cells and significantly fewer CD4^+^ T-cells and neutrophils than the *Itga11^+/+^* papillomas (**Figure 4F**). This result implies that the genetic depletion of α11 expression promotes the development of tumor-suppressive stroma in cSCC.

### Integrin α11 regulates LOX and PDGFRβ expression in CAFs

**Figure 5:**
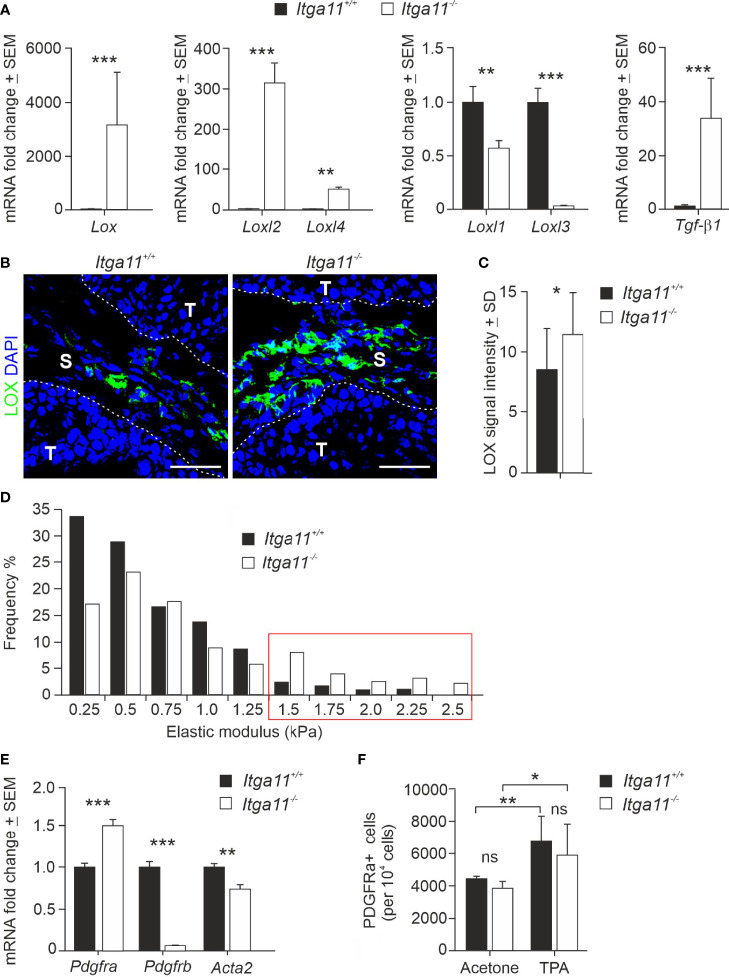
Expression of LOX family members and CAF markers in *Itga11^−/−^* skin tumors. **(A)** RT-qPCR analysis of lysyl oxidase (*Lox*), LOX-like enzymes (*Loxl1-4*), and transforming growth factor beta-1 (*Tgfβ1*) in *Itga11^+/+^* and *Itga11^-/-^* skin papillomas at week 20. The data are an average of ten samples (from different individuals) per genotype and normalized with endogenous *Gapdh*. **(B)** Representative immunofluorescence staining of LOX in *Itga11^+/+^* and *Itga11^-/-^* papillomas. LOX signals are prominent in α11-deficient skin tumors and widely distributed within the tumor stroma. Scale bars, 50 μm. S, stroma; T, tumor. **(C)** Quantification of LOX immunofluorescence in *Itga11^+/+^* and *Itga11^-/-^* papillomas. Twelve to 15 images from five tumors from five different individuals/genotype were quantified using Fiji ImageJ analysis software. **(D)** Tumor stiffness measurements by atomic force microscopy. Histograms of the Young’s elastic modulus (kPa) of the *Itga11^+/+^* (n = 4) and *Itga11^-/-^* (n = 3) papillomas collected at weeks 20–25. **(E)** RT-qPCR analysis of fibroblast markers PDGFRα, PDGFRβ, and αSMA (*Acta2*) in *Itga11^+/+^* and *Itga11^-/-^* papillomas collected at week 20. Data are an average of ten samples (from different individuals) per genotype. Values were normalized with *Gapdh*. **(F)** Numbers of Lin^-^ PDGFRα^+^ cells in the acetone-treated normal and TPA-treated hyperplastic skin of the *Itga11^+/+^* and *Itga11^-/-^* mice. In **(A, C, E, F)** *, p<0.05, **, p<0.01, ***, p<0.001. ns, not significant

We then deciphered the potential mechanisms that could explain the conspicuous changes observed in the collagenous matrix in α11-deficient skin tumors. Collagen assemblies are stabilized by covalent intra- and intermolecular crosslinks in collagen fibrils, which are predominantly catalyzed by lysyl oxidase (LOX) and four LOX-like enzymes (LOXL1-4) (44, 45). The RT-qPCR analysis showed a surprisingly high, on average 3,000-fold, increase in LOX transcripts in the *Itga11^−/−^* tumors in comparison with the *Itga11^+/+^* tumors (**Figure 5A**). Also, LOXL2 and LOXL4 mRNA levels were markedly upregulated in α11-deficient skin tumors, on average by 300-fold and 50-fold relative to the controls, respectively. In contrast, LOXL1 and LOXL3 mRNA levels were weakly downregulated in *Itga11^−/−^* tumors. The mRNA levels of TGFβ1, a key inducer of LOX family members, was also highly upregulated in *Itga11^−/−^* tumors relative to *Itga11^+/+^* tumors (**Figure 5A**). The immunofluorescence staining of mouse tumor tissues revealed prominent LOX signals in *Itga11^−/−^* papillomas, whereas there were clearly fewer in *Itga11^+/+^* papillomas. The LOX signals were distributed throughout the papilloma stroma and likely represented CAFs, while tumor cells were LOX-negative (**Figure 5B**). The quantification of stromal LOX signal intensities showed a significant difference between genotypes (**Figure 5C**). The large highly crosslinked collagen bundles observed in the *Itga11^−/−^* tumors are expected to lead to an increase in tissue stiffness. In agreement with this, atomic force microscopy measurements demonstrated a shift toward a higher elastic modulus in the α11-deficient skin tumors (**Figure 5D**).

We then addressed cell differentiation in *Itga11^−/−^* skin and skin tumors by analyzing the expression of various MSC and fibroblast markers in the knockout and control papillomas *via* qRT-PCR and by utilizing tissue immunostainings, FACS analysis, and *in vitro* cell cultures. We found that the mRNA levels of *αSMA* and *PDGFRβ*, markers of activated myofibroblasts and CAFs, were significantly lower in the *Itga11^−/−^* papillomas as compared with the *Itga11^+/+^* papillomas. In particular, *PDGFRβ* expression in knockout tumors was negligible as compared to that in control tumors. The MSC and pan-fibroblast marker *PDGFRα* showed significantly higher expression in the knockout tumors than in control tumors (**Figure 5E**).

Immunofluorescence demonstrated somewhat weaker PDGFRβ signals in the stroma of *Itga11^−/−^* tumors than in the *Itga11^+/+^* tumors, whereas αSMA signals were mostly alike between the two genotypes. However, the quantification of signal intensities did not reveal significant differences in PDGFRβ or αSMA expression between the *Itga11^−/−^* and *Itga11^+/+^* papillomas (**Supplementary Figures 4A, B**). Hyperplastic skin can be considered to represent an early step in skin tumor development (24) and was induced in *Itga11^−/−^* and *Itga11^+/+^* mice by repeated treatments of TPA. In comparison to the acetone-treated control skin, a notable increase was observed in the number of PDGFRβ−positive cells in the hyperplastic dermis of the *Itga11^+/+^* (**Supplementary Figure 4C**). In contrast, the number of PDGFRβ< mice cells increased only marginally in the hyperplastic skin of the *Itga11^−/−^* mice after the TPA treatments (**Supplementary Figure 4C**). The FACS analysis showed that the number of PDGFRα mice−lineage cells increased significantly upon TPA induction in both mouse strains but that their numbers were equal between genotypes, both in the healthy skin and in the inflamed skin (**Figure 5F**). These observations indicate that, in the mouse skin, α11β1 signalling affects myofibroblast activation, and especially the differentiation of the PDGFRβ−positive subpopulation, both in TPA-induced epidermal hyperplasia and chronic inflammation and during the cSCC development in the DMBA/TPA model.

Finally, to further shed light on the roles of α11β1 signaling in MSCs and address the previous findings regarding the accumulation of adipocytes in *Itga11^−/−^* skin wounds (46), we compared the differentiation potential of SVF progenitors from the knockout and control mice under *in vitro* culture conditions. This assay did not reveal differences in adipocyte differentiation between the genotypes, as assessed by cell morphology, Oil-Red-O staining of the lipid content, and mRNA expression of the adipogenic markers peroxisome-proliferator activated receptor gamma (*Pparγ*) and adipocyte protein 2 (*aP2*) (**Supplementary Figure 5**). Our current immunostainings of tumor tissues and FACS analysis, together with the previously published data on α11 in fibroblasts, reinforce the perception that integrin α11β1 signaling plays an active role in mediating the differentiation of skin fibroblasts into myofibroblasts and CAFs but does not play a significant role in the differentiation of SVF progenitors into subcutaneous adipocytes.

## Discussion

Substantial evidence has been gathered to demonstrate the relevance of α11β1 signaling in CAFs in distinct solid cancer types, as well as in myofibroblasts during wound healing [reviewed in (4, 11)]. Here, we report our novel findings on the notable upregulation of integrin α11 expression in the stromal compartment of human and mouse cSCCs, as well as the significant impairment of DMBA/TPA-induced skin tumor growth in mice, accompanied by interesting alterations in the tumor stroma, in the absence of α11 integrin.

The α11 expression was most prominent in the desmoplastic stroma of malignant high-grade human cSCCs, where the intense, tangle-like α11 signals resembled the staining patterns that have previously been reported for this integrin subunit in human HNSCC (15), as well as in human breast, lung and pancreatic adenocarcinomas (12, 16, 17). The quantification of immunosignals showed that α11 expression was significantly higher in cSCC in the *in situ* stage than in benign and premalignant skin lesions, which suggests that α11 could potentially be utilized as a novel early biomarker to improve the diagnosis and prognostication of this common human cancer. However, using our current, limited cSCC material, we were unable to evaluate whether α11 can distinguish cases with a high risk of cSCC progression and metastasis from those that involve less aggressive tumors and can be treated surgically. Our ongoing work exploiting large and well-characterized cSCC cohorts with full clinical data (47) will better reveal the value of α11 as a novel biomarker in cSCC.

Recent scRNA-seq and bulk RNASeq analyses have identified integrin α11 among the upregulated genes in CAF subpopulations in several human carcinomas, including HNSCC, cutaneous melanoma, lung cancer, breast cancer, and PDAC (40, 48–50). These CAF signatures are associated with active TGFβ signaling and matrix synthesis and remodeling and, in addition to α11, include transcripts for *PDGFRB, ACTA2, FAP, LOX*, periostin, fibronectin, and various collagens, just to mention a few. Thus far, the data on CAF signatures in non-melanoma skin cancers are limited, but one study demonstrated the upregulation of a prominent number of genes involved in ECM remodeling in human basal cell carcinoma (BCC) (51). Interestingly, *ITGA11* was found among these upregulated CAF genes, together with *PDGFRB, COL1A1, COL1A3, LOXL2* and *P4HA*, which all were downregulated in our cSCC mouse model with *Itga11* depletion. The findings published by Omland and coworkers highlight the important role of the microenvironment and CAF activity in the regulation of BCC and point to a tumor-promoting signalling axis, which links integrin α11 with CAF activation and ECM remodeling in this skin cancer type. Another study showed that specific CAF phenotypes can cluster cutaneous melanomas, BCCs and cSCCs into distinct histological subgroups and that this clustering can be used to facilitate diagnosis and even predict tumor progression. In this analysis, cSCC was characterized by the high expression of CAF and epithelial-to-mesenchymal (EMT) markers PDGFRβ, αSMA, podoplanin, Zeb1, Slug, and Twist (52).

With respect to cSCC, next-generation sequencing efforts have mainly targeted transcriptional profiles and genomic alterations in carcinoma cells, but one recent study reports scRNA-seq data on various TME cell types in cSCC and also lists the top ligand-receptor pairs between tumor-specific keratinocytes and CAFs (53). *ITGB1* and *ITGA1* were found among the key CAF receptors that mediate crosstalk between tumor cells and CAFs by interacting with *COL1A1* and *TNC* and, thereby, modulating the TME. Although *ITGA11* was not found in this scRNA-seq analysis, our expression and mouse data predict similar functions on the part of α11β1 in regulating the CAF-tumor cell interplay in cSCC.

Our previous analysis of open databases showed that *ITGA11* expression was correlated positively with *PDGFRB*, *ACTA2*, *TNC*, *COL1A1*, and *COL1A3* in human breast cancer, and the immunostainings showed that α11, PDGFRβ, and TNC were colocalized to CAF subsets in human breast cancer stroma (16). By subjecting wild-type and α11-deficient CAFs from PyMT mouse mammary tumors io 3D spheroid assays, we showed that the interaction between α11 and PDGFRβ in CAFs is needed for efficient PDGFRβ signaling through JNK upon PDGF-BB stimulation. PDGFRβ activation then leads to ECM remodeling, including TNC upregulation, and promotes CAF migration and CAF-induced tumor cell invasion (16).

Our novel observation regarding reduced PDGFRβ and TNC expression in α11-deficient mouse skin tumors is in line with these previous findings on breast cancer and suggests that the α11/PDGFRβ/TNC axis may promote also skin carcinogenesis. Our attempts to establish CAF lines from *Itga11^-/-^* skin tumors using either the explant method or anti-PDGFRα antibody-based FACS were not successful, and thus, the functions and molecular mechanisms involving α11, PDGFRβ and TNC could not be studied further under *in vitro* conditions. Nevertheless, our findings on reduced PDGFRβ expression in the absence of α11, not only in *Itga11^-/-^* skin tumor stroma but also in chronically inflamed dermis, suggest the need for further studies on integrated α11β1 and PDGFRβ signaling. In fact, an early study showed that PDGFRβ is highly expressed in fibroblasts in skin biopsies of systemic sclerosis (SS) patients (54), and a recent scRNA-seq analysis identified *ITGA11* as enriched in SS skin myofibroblasts (55). Interestingly, the blocking of fibrotic activity in skin SS myofibroblasts with a YAP inhibitor, verteporfin, reduced the expression of *ITGA11, ITGB1, PDGFRB*, *COL1A1*, and many other ECM-associated genes (56), further reinforcing the view that α11 and PDGFRβ are concomitantly regulated and form a signaling hub that promotes myofibroblast activation in fibrotic tissues.

An unexpected observation in our study was the obvious alteration of the collagenous stroma in the *Itga11^−/−^* skin tumors. Despite the apparent defect in the activation of the matrix-producing CAF subtype in the knockout tumors, the collagen fibers in the α11-deficient tumor stroma were conspicuously abundant, large, and parallelly assembled, as compared to the less organized and more scattered collagen fibers in control tumors. This finding is likely due to the high expression of LOX, LOXL2, and LOXL4 in *Itga11^−/−^* tumors, driven by noticeable TGFβ1 upregulation, while LOXL1 and LOXL3 were slightly downregulated in *Itga11^−/−^*. A recent study showed a strong correlation between CAF-expressed α11 and LOXL1 expressions in lung adenocarcinoma, and LOXL1 was shown play a critical role in inducing matrix remodeling and collagen fiber alignment, thereby supporting tumor growth and progression in a xenograft model (14). Similar to our observations in *Itga11^−/−^* skin tumors, LOXL1 expression was decreased in *Itga11^−/−^* mouse embryonic fibroblasts. The findings by Zeltz and coworkers in lung adenocarcinoma and by us in cSCC demonstrate a link between α11 and ECM crosslinking by LOX and LOXL enzymes but also imply that the molecular mechanisms whereby α11β1 reorganizes the ECM vary by tumor and CAF subtype.

It is well established that TGFβ signaling plays dual roles in cancer, including in cSCC (57, 58). In DBMA/TPA-treated mouse skin, TGFβ1 is upregulated in basal cells in papillomas and inhibits cell proliferation and papilloma formation, whereas carcinoma cells are devoid of TGFβ1 expression. However, the upregulation of TGFβ1 in macrophages and fibroblasts enhances malignant transformation and metastasis in the later stages of skin carcinogenesis (57). Hence, our data indicating significantly higher levels of TGFβ1 in *Itga11^-/-^* papillomas than in *Itga11^+/+^* papillomas suggest that enhanced TGFβ1 signaling could be one reason for the reduced tumor cell proliferation and impaired primary tumor growth. TGFβ1 expression in fibroblasts, in turn, promotes CAF differentiation in an autocrine manner, leading to the increased deposition of ECM proteins, desmoplasia and tissue stiffening (59). TGFβ1 induces α11 expression in skin myofibroblasts during wound healing (46), and we observed higher levels of TGFβ1 in *Itga11^-/-^* skin tumors as compared to *Itga11^+/+^* tumors. However, according to our data, the *Itga11^-/-^* skin tumor CAFs do not respond as efficiently to TGFβ1 induction as the control CAFs, as demonstrated by the downregulation of fibrillar collagen and TNC biosynthesis in the knockout tumors. The high levels of LOX, LOXL2, and LOXL4 transcripts in α11-deficient CAFs are congruent with the observed formation of extensive, linear collagen bundles and a shift toward stiffer skin tumor tissue in this mouse strain.

Elevated fibrillar collagen synthesis, crosslinking and fiber alignment are usually associated with increased cancer invasion and metastasis and poor patient outcome, as exemplified by pioneering studies on breast cancer (60, 61) and PDAC (62). However, recent data have challenged this view by showing, for example, that the deletion of either the αSMA-expressing myCAF subset or the depletion of all hepatic stellate cell-derived CAFs in PDAC mouse models decreases tumor growth and metastasis significantly (63, 64). Bhattacharjee et al. linked the tumor-promoting effects of CAFs with high expression levels for hyaluronan in myCAFs and hepatocyte growth factor in iCAFs. Moreover, tumor progression may be opposed by myCAF-synthesized fibrillar collagen I, which restrains tumor spread mechanically and, at the same time, suppresses the stiffness-induced mechanosignals from the ECM (64). In agreement with this novel experimental data, a previous work with PDAC patient samples showed that, in the absence of αSMA-positive CAFs, collagen deposition is correlated with a good prognosis (65). Considering these data regarding PDAC, our observations regarding the defects in the activation of matrix-producing CAFs, LOX upregulation, and the formation of dense and aligned collagen matrix and the impaired growth of skin tumors in *Itga11^-/-^* mice are not necessarily contradictory. In contrast, our data highlight the extreme complexity of TME, CAF, and ECM functions in tumors, as well as the central role of α11β1 signaling in CAFs, and also point to differences in their interplay in different tumor types. The dynamic interactions and crosstalk between different stromal cell types and the insoluble matrix in the TME have important immune modulatory functions in tumors, as are also evidenced by our observation regarding altered immune cell profiles in the α11-deficient skin tumors. We postulate that the effects of α11β1 signaling on the immune environment are indirect because both CAF-immune cell interactions and ECM deposition and collagen assembly are known to significantly affect immune cell recruitment and phenotypes (66–69).

In summary, we describe here, for the first time, the expression of α11 integrin in the stroma of human and mouse cSCC and show that α11β1 signaling in CAFs promotes skin carcinogenesis in a chemical mouse model. We conclude that α11β1 operates in a subset of skin tumor CAFs, likely in co-operation with PDGFRβ signaling, thereby regulating ECM synthesis and collagen assembly to encourage cSCC growth and progression. Further studies with sophisticated experimental models are needed to reveal the molecular mechanisms of α11β1 in skin tumor CAFs, as well as its role in the interplay of CAFs with other cell types in the TME in cSCC. Because α11 is not essential for skin development, it should be evaluated as a therapeutic target in skin cancer.

## Data availability statement

The raw data supporting the conclusions of this article will be made available by the authors, without undue reservation.

## Ethics statement

The studies involving human participants were reviewed and approved by Finnish National Supervisory Authority for Welfare and Health, The Ethical Committee of the Northern Ostrobothnia Hospital District. The patients/participants provided their written informed consent to participate in this study. The animal study was reviewed and approved by The National Animal Experiment Board of Finland, Regional State Administrative Agency.

## Author contributions

Conceptualization: RH, DG, and TPi. Methodology: GM-N, H-RT, RH, GW, TPe, SM-K, JK, and JM. Investigation: GM-N, H-RT, NP, VI, RD, HL, TPe, KK and JK. Writing − Original draft: GM-N and H-RT. Writing − Review and Editing: RH, DG, VI, JM, and TPi. Visualization: GM-N, H-RT, and RH. Supervision: RH, DG, S-MK, HR, and TPi. Funding Acquisition: TPi, RH, DG, H-RT, and GW. All authors contributed to the article and approved the submitted version.

## Funding

The research leading to these results has received funding from the People Programme (Marie Curie Actions) of the European Union’s Seventh Framework Programme FP7/2007-2013/under REA grant agreement no. 316610 (for GM-N and HL) and from the Academy of Finland (grants 308867 and 284065 for TPi), the Cancer Foundation Finland (grants 190147 and 170138 for TPi), the Sigrid Jusélius Foundation (TPi and RH), the Jane and Aatos Erkko Foundation (TPi), the Norwegian Centre of Excellence grant from the Research Council of Norway (ID 223250) (DG), the Western Norway Regional Health Authority (ID 911899) (DG), the National Science and Engineering Research Council of Canada (GW), the Finnish Medical Society Duodecim (H-RT and NP), the Finnish Medical Foundation (H-RT and NP), and the Kerttu Saalasti Foundation (TPe).

## Acknowledgments

We thank Päivi Tuomaala, Erja Tomperi, and Jaana Peters for their excellent technical assistance; Raija Sormunen and Ilkka Miinalainen for electron microscopy analyses; and Tamara Monteagudo Aboy for her contribution on human sample analysis. This work was carried out with the support of the Oulu Laboratory Animal Centre, Biocenter Oulu Research Infrastructures and Biocenter Finland.

## Conflict of interest

The authors declare that the research was conducted in the absence of any commercial or financial relationships that could be construed as a potential conflict of interest.

## Publisher’s note

All claims expressed in this article are solely those of the authors and do not necessarily represent those of their affiliated organizations, or those of the publisher, the editors and the reviewers. Any product that may be evaluated in this article, or claim that may be made by its manufacturer, is not guaranteed or endorsed by the publisher.
