# Supplementary material for: Upregulated integrin α11 in the stroma of cutaneous squamous cell carcinoma promotes skin carcinogenesis
Source: Front Oncol. 2022 Aug 8;12:981009. doi: 10.3389/fonc.2022.981009 (PMC9393502; doi:10.3389/fonc.2022.981009)
Supplement: Supplementary file 1 [file DataSheet_1.pdf]

Supplementary Materials

Supplementary Table 1. Primary antibodies used for immunofluorescence staining of mouse tissues

Antibody	Manufacturer	Reference	Dilution
anti-human integrin α11	In house	Smeland et al. 2020	1:1000
anti-mouse integrin α11	In house	Velling *et al.* 1999	1:200
anti-BrdU	Sigma-Aldrich	B2531	1:200
anti-cytokeratin 5 (CK5)	Covance	PRB-160P	1:100
anti-αSMA-Cy3	Sigma-Aldrich	C6198	1:200
anti-NG2	Sigma-Aldrich	AB5320	1:50
anti-Ki67	Dako	M7249	1:25
anti-LOX	Novus Biologicals	NB100-2527	1:500
anti-PDGFRβ (CD140b)	Thermo Fischer	14-1402-82	1:50

Supplementary Table 2. Antibodies used for fluorescence-activated cell sorting (FACS)

Antibody	Manufacturer	Reference
CD11b	BD Biosciences	553311
Lineage cocktail (Lin) (APC)	BD Biosciences	558074
Ly-6A/E (Sca-1)	BD Biosciences	558162
Ly-6C	BD Biosciences	553104
Ly-6G	BD Biosciences	560601
Mouse T Lymphocyte Subset Antibody Cocktail	BD Biosciences	558391
PDGFRα	BD Biosciences	562776

Supplementary Table 3. Primer sequences used in qRT-PCR

Gene	Forward primer (5' to 3')	Reverse primer (5' to 3')
Acta2	GATCAAGATCATTGCCCCT	GTGCTAGAGGCAGAGCAGGGG
Pdgfra	CTTCGGAAGAGAGTGCCATC	CACCAGGTCCGAGGAATCTA
Pdgfrb	GGAGATTCGCAGGAGGTCAC	ATAGCGTGGCTTCTTCTGCC
Tnc	CAGTGGAGTACGAGCTGCAT	TAAACTTGGTGGCGATGGTA
Tgfb1	GAGCCCGAAGCGGACTACTA	TGGTTTTCTCATAGATGGCGTTG
Col1a1	TCCTGCCGATGTCGCTATC	CAAGTTCCGGTGTGACTCGTG
Col3a1	CCACGAGGTGACAAAGGTGA	GCCAGGGAATCCTCGATGT
Loxl1	GAGTGCATATTGCGCTTCCC	GGTTGCCGAAGTCACAGGT
Loxl2	CAGAGAAGACCTACAACCCCA	AGTGCCCGTGCAGTTCATAG
Loxl3	TGTGACAGAATGCGCCTCTC	ACCTCAATGACGTTGGAGTCT
Loxl4	GGACTATATCTTCCAGGTGGTTGT	GCACCGTATCATGTTATTGGAGAA
P4ha1	AAAACCAAGGCTGAGGCGAGC	GCCTGCCGTCTGTAAGCCCAC
P4ha2	GACGCCGGACTGGCCACC	AGCTTCTTCTGCCTCCGGGGT
Gapdh	AACTTTGGCATTGTGGAAGG	GGATGCAGGGATGATGTTCT

Supplementary Data

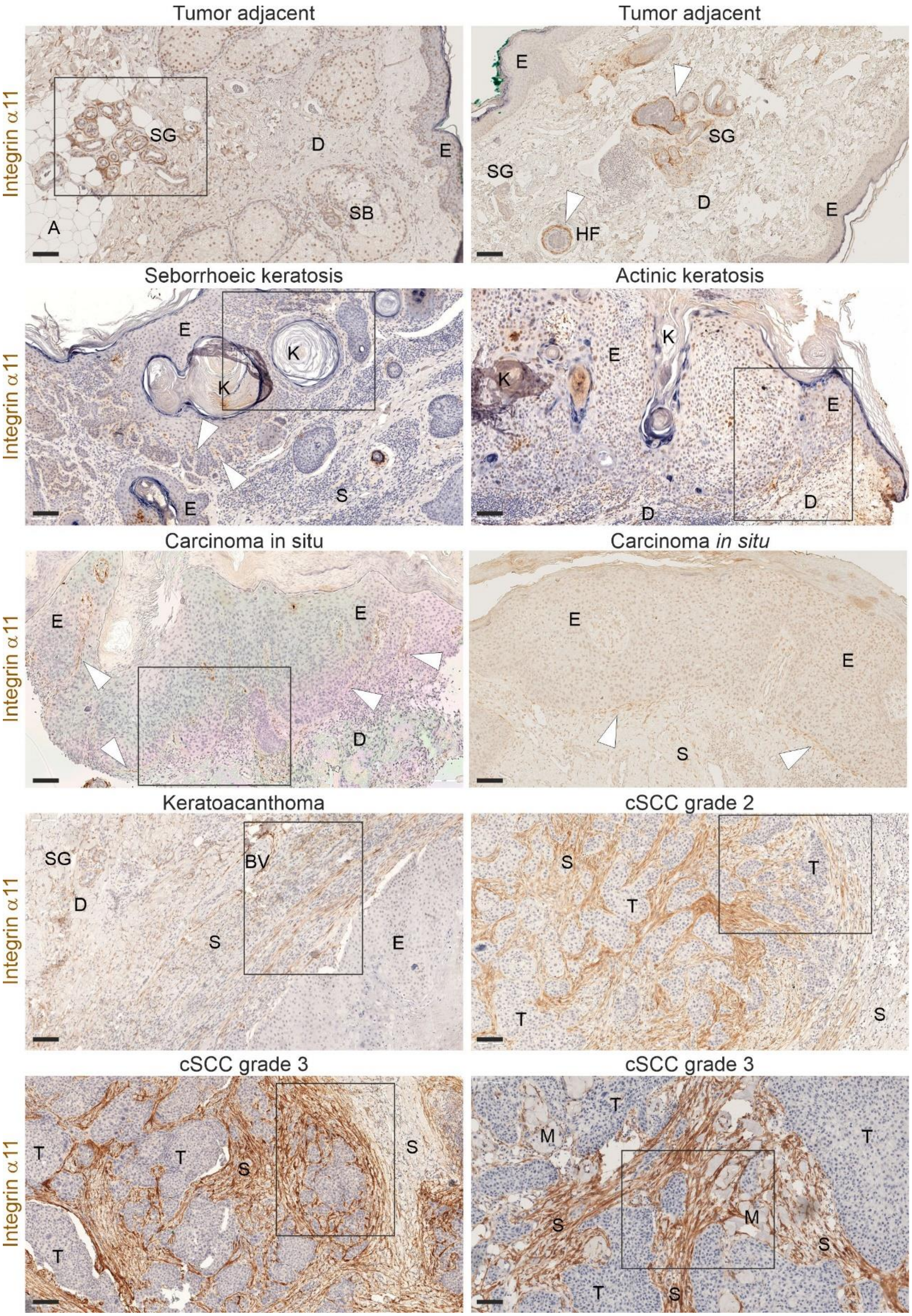

Supplementary Figure 1. Expression and localization of integrin α11 in human cutaneous lesions. Large images of immunohistochemical staining for integrin α11 in tumor-adjacent normal skin, seborrhoeic keratosis, premalignant actinic keratosis, squamous carcinoma *in situ*, keratoacanthoma, and cutaneous squamous cells carcinomas (cSCC) of different grades. The areas presented in the main Figure 1 are indicated. Arrowhead marks α11-positive cells. Abbreviations: A, adipocyte; BV, blood vessel; D, dermis; E, epidermis; HF, hair follicle; K, keratin; M, muscle; S, stroma; SB, sebaceous gland; SG, salivary gland; T, tumor. Scale bars, 100 μm.

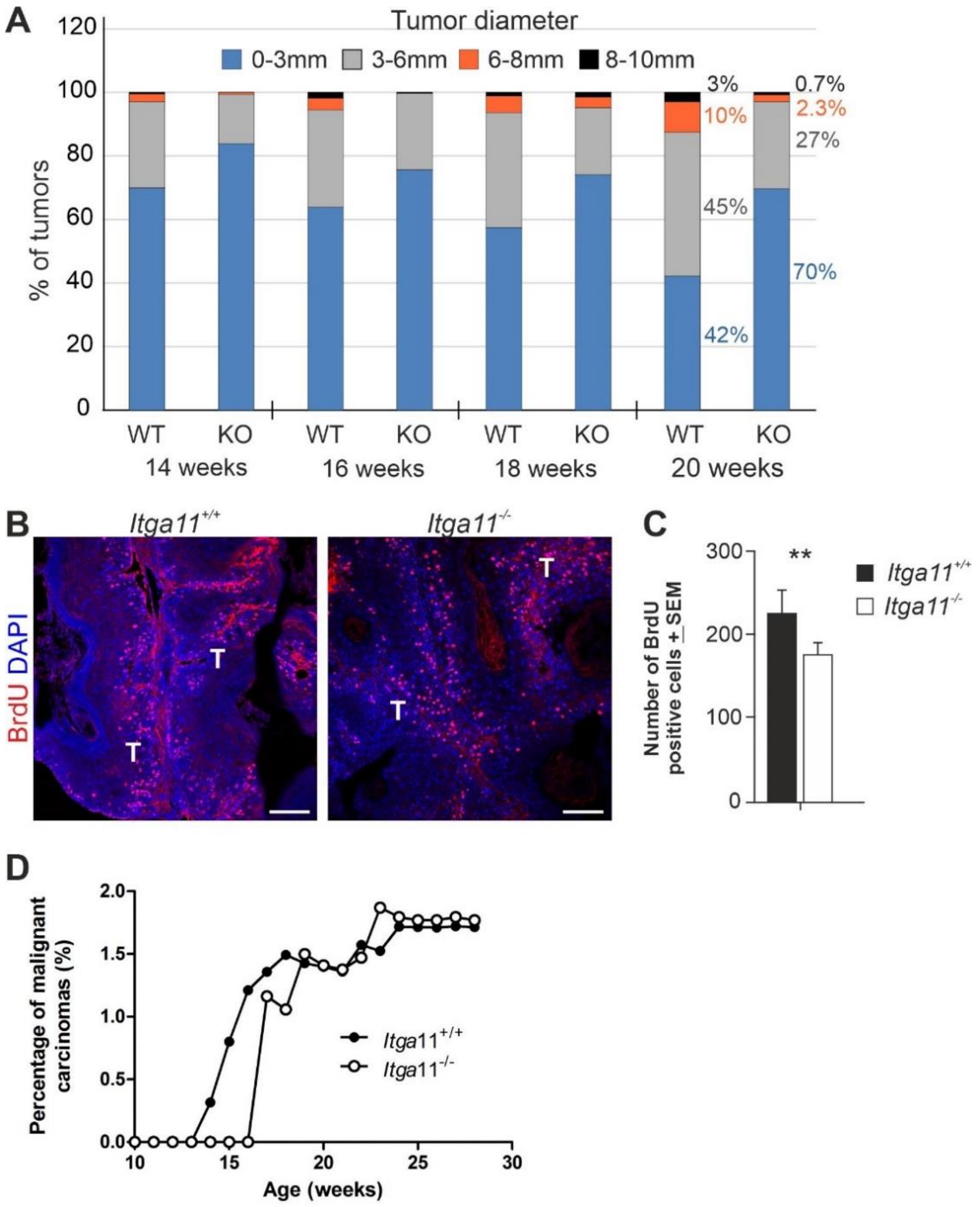

Supplementary Figure 2. Integrin α11 regulates skin tumorigenesis in the DMPA/TPA model. (A) Tumor size in the *Itga11*$^{+/+}$ (WT; n = 25) and *Itga11*$^{-/-}$ (knockout, KO; n = 21) mice. Skin tumors are stratified into four categories by tumor diameter. The proportion of tumors with a diameter over three mm is larger in the *Itga11*$^{+/+}$ mice at all time points. At week 20, for example, 70% of knockout tumors fall in the 0–3 mm group, and only 2.3% fall in the 6–8 mm group, whereas 42% of control tumors belong to the 0–3 mm group, and 10% belong to the 6–8 mm group. **(B-C)** Tumor cell proliferation. Proliferating cells were labelled with BrdU two hours before euthanasia and detected in tumor specimens with an anti-BrdU antibody. **(B)** Representative images of anti-BrdU staining of *Itga11*$^{+/+}$ and *Itga11*$^{-/-}$papillomas. Scale, 100 μm. T, tumor. **(C)** Quantification of BrdU-positive cells. Ten papillomas per genotype (from different mice) and 4–5 microscopic fields for each sample at a magnification of 200× were counted. **(D)** Malignant conversion of benign papillomas to malignant cSCCs based on macroscopical scoring. The frequency of malignant conversion did not vary between genotypes.

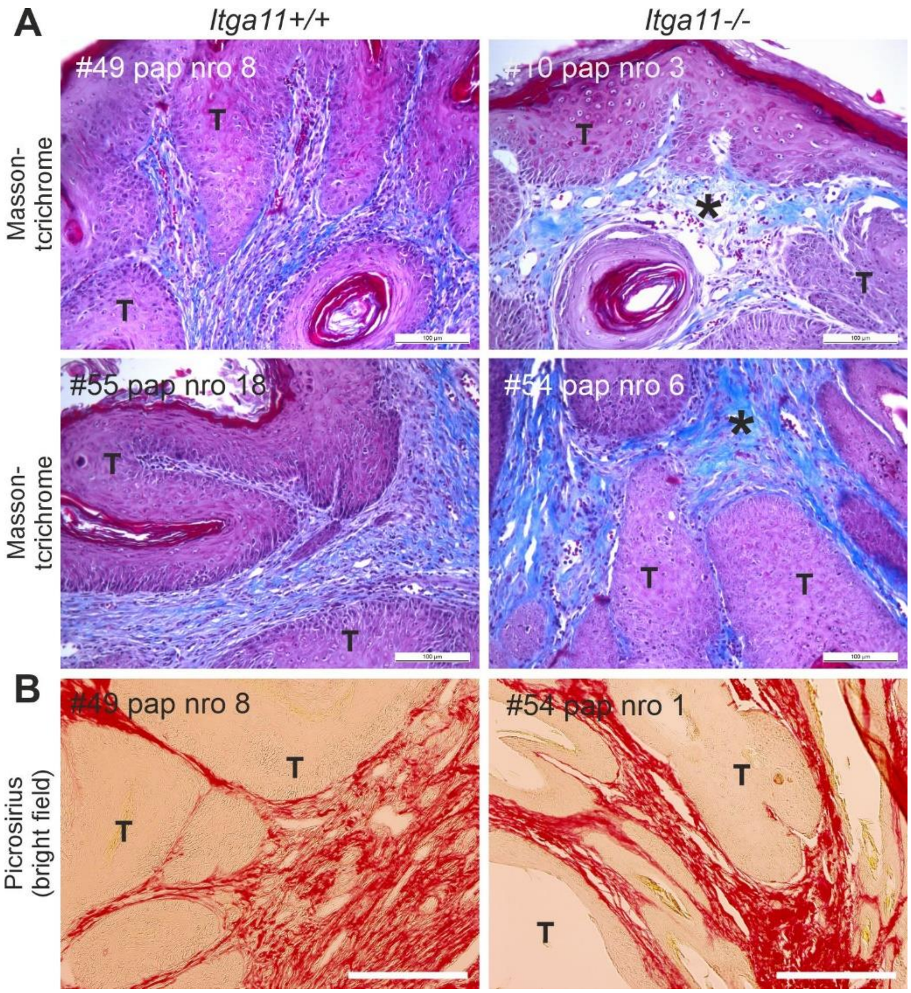

Supplementary Figure 3. Stromal collagen *Itga11*$^{+/+}$ and *Itga11*$^{-/-}$ papillomas. (A) Representative example images of Masson trichome-stained papillomas from two *Itga11*$^{+/+}$ and two *Itga11*$^{-/-}$ mice. **(B)** Picrosirius red-stained papillomas imaged with bright field light microscopy. The same samples imaged under polarized light are shown in the main Figure, panel 4C. Scale bars, 100 μm. T, tumor; asterisks in A, hyalinized collagen.

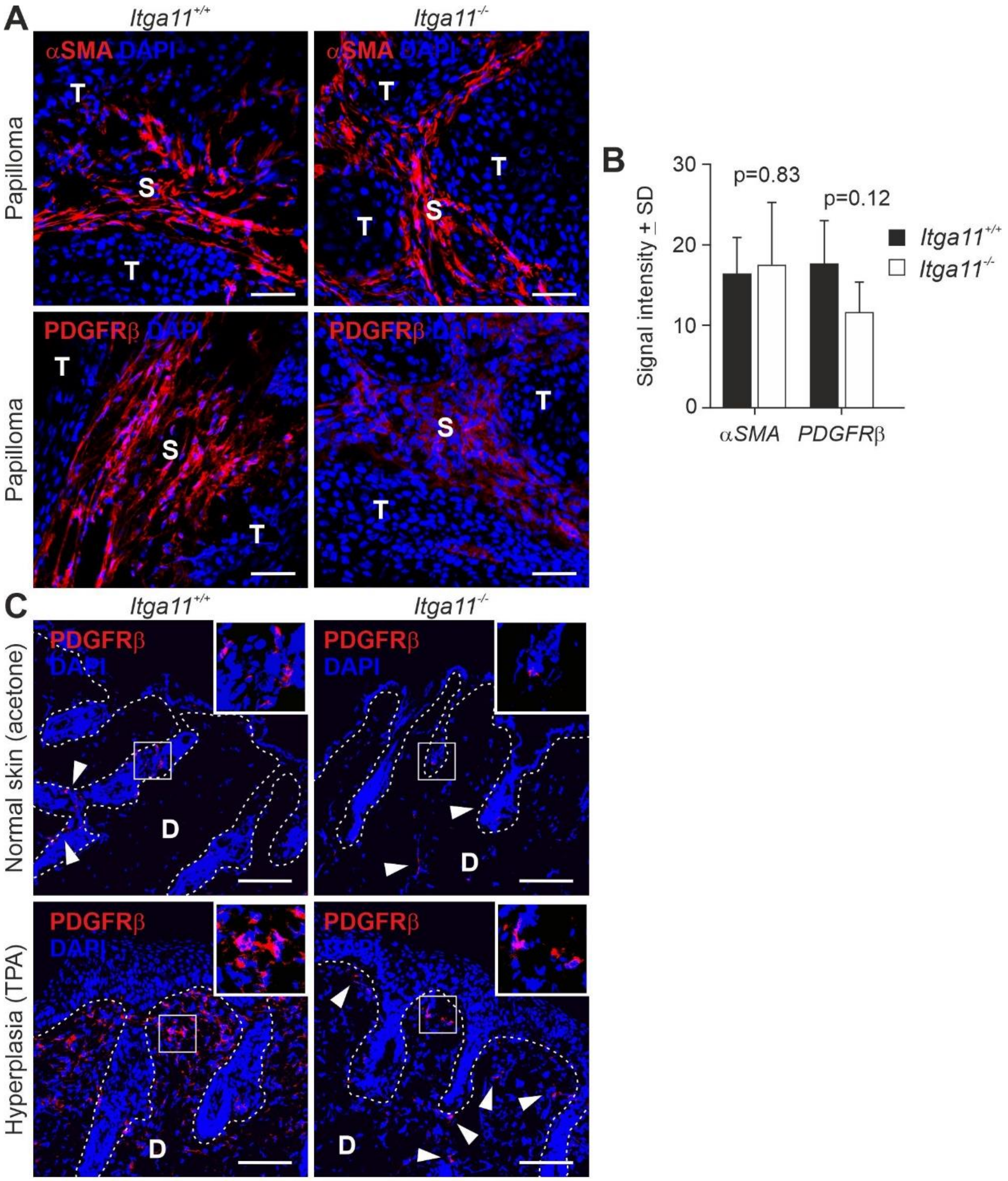

Supplementary Figure 4. Immunofluorescence staining of CAF/myofibroblast markers in mouse papillomas and hyperplastic skin. (A) Expression and localization of αSMA and PDGFRβ in the stroma of papillomas. **(B)** Quantification of αSMA and PDGFRβ immunofluorescence in *Itga11*$^{+/+}$ and *Itga11*$^{-/-}$ papillomas. Ten to 15 images from three (for αSMA) and five (for PDGFRβ) tumors per genotype (from different individuals) were quantified using Fiji ImageJ analysis software. p-values are shown in the image. **(C)** PDGFRβ staining of acetone-treated normal and TPA-treated hyperplastic skin specimens of the *Itga11*$^{+/+}$ and *Itga11*$^{-/-}$ mice. PDGFRβ is clearly induced in the *Itga11*$^{+/+}$ dermis upon TPA treatment but not in the *Itga11*$^{-/-}$ dermis. In both genotypes, PDGFRβ signals localize close to hair follicles in the healthy skin, whereas they localize mainly in dermal cells in the hyperplastic skin (arrowheads, inserts). In A and C, Scale bars, 100 μm; D. dermis; S, stroma; T, tumor**.**

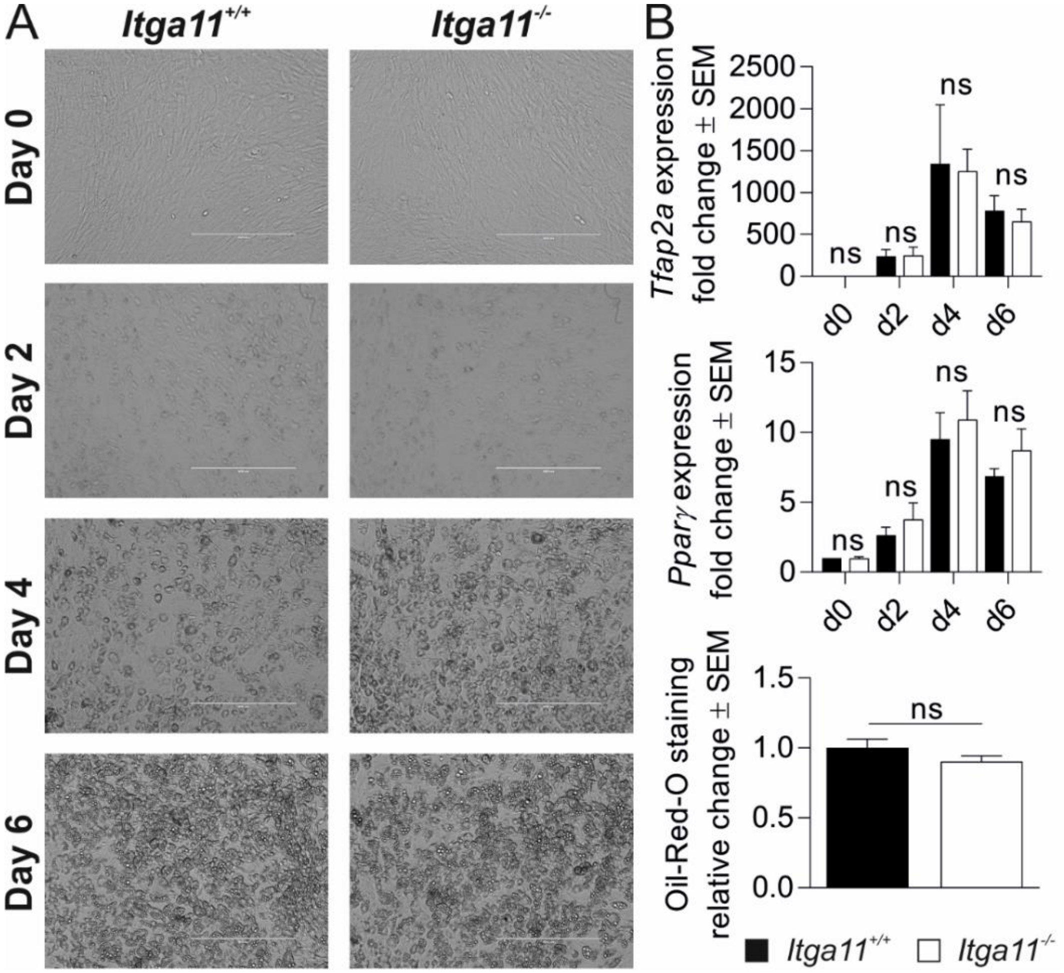

Supplementary Figure 5. *In vitro* adipogenesis assay. Mesenchymal progenitors were isolated from the inguinal white adipose tissue of the *Itga11*$^{+/+}$ and *Itga11*$^{-/-}$ mice and seeded on culture plates. The number of isolated cell batches was 3–4 per genotype. Adipogenic differentiation was induced with a cocktail consisting of 5µg/ml insulin, 1µM rosiglitazone, 0.5mM isobutylmethylxanthine, and 1µM dexamethasone and followed for six days. **(A)** Representative images of cell cultures at indicated time points. Scale, 400 µm. **(B)** RT-qPCR for the adipogenesis markers Ap2 (*Tfap2a*) and PPARγ (*Pparg*) showed no differences in adipocyte differentiation between the *Itga11*$^{+/+}$ and *Itga11*$^{-/-}$ cells. The quantification of Oil-Red-O staining for the lipid content in adipocytes showed no differences between genotypes.

Supplementary Table 4. Histopathological analysis of mouse cSCC suspects.

	Itga11 $^{+/+}$	***Itga11*** $^{-/-}$
Number of mice (>15 weeks in experiment)	22	18
Total number of macroscopic cSCC suspects	14	7
**Histological analysis and grading of cSCC suspects n (%)**		
cSCC Grade 1	3	1
cSCC Grade 2	3	1
cSCC Grade 3	3	0
**Total cSCC**	**9 (64%)**	**2 (29%)**
Benign papilloma	2	2
Dysplastic papilloma	2	3
Cystic papilloma	1	0
**Total papilloma**	**5 (36%)**	**5 (71%)**
